# The Actinome of *Dictyostelium discoideum* in Comparison to Actins and Actin-Related Proteins from Other Organisms

**DOI:** 10.1371/journal.pone.0002654

**Published:** 2008-07-09

**Authors:** Jayabalan M. Joseph, Petra Fey, Nagendran Ramalingam, Xiao I. Liu, Meino Rohlfs, Angelika A. Noegel, Annette Müller-Taubenberger, Gernot Glöckner, Michael Schleicher

**Affiliations:** 1 Adolf Butenandt Inst./Cell Biology and Center for Integrated Protein Science (CIPSM), Ludwig-Maximilians-University, Muenchen, Germany; 2 dictyBase, Center for Genetic Medicine, Northwestern University, Chicago, Illinois, United States of America; 3 Department of Biology II, Ludwig-Maximilians-University, Muenchen, Germany; 4 Institute for Biochemistry I, Center for Molecular Medicine Cologne (CMMC) and Cologne Excellence Cluster on Cellular Stress Responses in Aging-Associated Diseases (CECAD), University of Cologne, Koeln, Germany; 5 Leibniz-Institute for Age Research - Fritz Lipmann Institute, Jena, Germany; University of Birmingham, United Kingdom

## Abstract

Actin belongs to the most abundant proteins in eukaryotic cells which harbor usually many conventional actin isoforms as well as actin-related proteins (Arps). To get an overview over the sometimes confusing multitude of actins and Arps, we analyzed the *Dictyostelium discoideum* actinome in detail and compared it with the genomes from other model organisms. The *D. discoideum* actinome comprises 41 actins and actin-related proteins. The genome contains 17 actin genes which most likely arose from consecutive gene duplications, are all active, in some cases developmentally regulated and coding for identical proteins (Act8-group). According to published data, the actin fraction in a *D. discoideum* cell consists of more than 95% of these Act8-type proteins. The other 16 actin isoforms contain a conventional actin motif profile as well but differ in their protein sequences. Seven actin genes are potential pseudogenes. A homology search of the human genome using the most typical *D. discoideum* actin (Act8) as query sequence finds the major actin isoforms such as cytoplasmic beta-actin as best hit. This suggests that the Act8-group represents a nearly perfect actin throughout evolution. Interestingly, limited data from *D. fasciculatum*, a more ancient member among the social amoebae, show different relationships between conventional actins. The Act8-type isoform is most conserved throughout evolution. Modeling of the putative structures suggests that the majority of the actin-related proteins is functionally unrelated to canonical actin. The data suggest that the other actin variants are not necessary for the cytoskeleton itself but rather regulators of its dynamical features or subunits in larger protein complexes.

## Introduction

Actin is an abundant and highly conserved globular protein (G-actin) that forms by polymerization into actin filaments (F-actin) a three-dimensional network, the general principle of the actin cytoskeleton in all non-muscle cells [1], [2], [3]. Dynamic rearrangements of the microfilament system determine the cell shape, provide mechanical support for contraction, enable cell movements, and participate in cell junctions. The number of actin genes varies drastically among eukaryotic organisms. Whereas *Saccharomyces cerevisiae* harbors only one gene that codes for a conventional actin, mouse contains 35, and the plant *Arabidopsis thaliana* 10 actin genes [4], [5]. In higher organisms actin isoforms are classified based on cell type and location, their isoelectric points and their amino acid sequences. Human actins are either specific for cardiac, skeletal or smooth muscle tissues, or are found in the cytoplasmic fraction only [6], [7]. It is still a mystery why nearly identical isoforms have to be so specific in their localization. The picture became even more complex after the discovery of actin-related proteins (Arps) [8]. Some of them are known to play an important role in actin polymerization (e.g. Arp2/3) or in chromatin remodeling (e.g. Arp 4, Arp 8), however others are with regard to their function still white spots on the map. The availability of many fully sequenced genomes allows now thorough analyses of important and ubiquitous protein families, and to draw conclusions for in vivo functions of yet uncharacterized proteins.

*Dictyostelium discoideum* is a soil amoeba which is able to undergo development and cell differentiation upon removal of nutrients [9]. The genome has a size of 34 MB organized in six chromosomes, it is very A/T-rich and codes for about 12,500 proteins including dozens of conventional actins and actin-related proteins [10]. *D. discoideum* is the best studied member of the Dictyostelidae, a large family of social amoebae that are at the evolutionary boundary from uni- to multicellular organisms. The family can be divided into four taxonomical groups which all share extensive cell migration during development and actin-based motile activities [11], [12]. Due to the relatively large number of actin genes, the *D. discoideum* genome provides a very good basis to study the ‘actinome’ for potential cellular targets and conserved sequence motifs. A comparison of these data with genomes from other Dictyostelidae as well as higher organisms up to mammals leads not only to a classification of the actin genome of *D. discoideum* but highlights also general features of actins and actin-related proteins in all eukaryotes.

## Results and Discussion

### I. The actinome of *D. discoideum*

#### Gene organization

**Figure 1:**
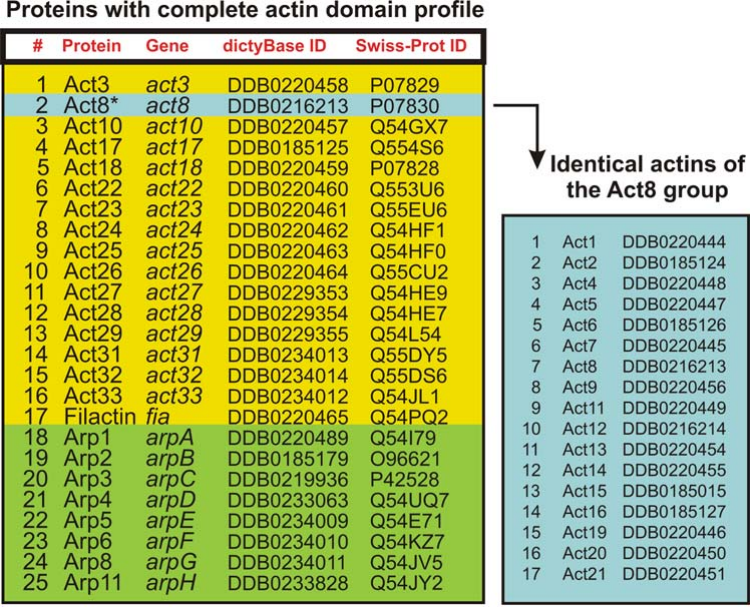
The *D. discoideum* actinome. 41 members of the actinome were identified according to their actin sequence profile. 17 conventional actins share identical amino acid sequences but are encoded by 17 distinct genes (Act8 group, right panel). 17 actins share high homologies to conventional actin but are different in their protein sequences (left panel, #1, 3–17). Nine members of the actinome are conserved actin-related proteins (Arps, #18–25).

The members of the *D. discoideum* actinome were identified according to their ‘actin sequence profile’. These profiles were based on multiple sequence alignments and profile-hidden Markov models from the ‘Pfam’ protein family database [13]. The *D. discoideum* actinome comprises 41 actins and actin-related proteins. Most interestingly, 17 conventional actins share identical amino acid sequences and thus form a functional group. These identical actins are encoded by 17 distinct genes. The other genes code for actin variants which differ in their amino acid sequences. These differences range between minor changes, e.g. only one D2E substitution in Act10, up to 295 non-identical amino acids in Act33. We identified three proteins in the *D. discoideum* actinome (DDB0234012, DDB0234013, DDB0234014) that have not been reported before [10]. Eight proteins with a characteristic actin sequence profile are homologues to known actin-related proteins (Arps, Fig. 1).

The *D. discoideum* genome contains seven potential actin pseudogenes (Table 1). Gene DDB0237409 which was originally listed as coding for the conventional actin Act30 [14], has now been verified and confirmed as a pseudogene. Two bases were deleted and caused a frame shift after codon 98. The other actin pseudogenes are very short and the homology to actin extends over a length between 41 and 135 amino acids only.

**Table 1:** Actin pseudogenes in the *D. discoideum* genome (aa: amino acids).

#	dictyBase ID	Gene	characteristics and maximal coding region
1	DDB0237409	*act30_ps*	frame shift, two ORFs with 106 aa and 258 aa
2	DDB0237453	*act34_ps*	partial actin domain with 41 aa
3	DDB0237452	*act35_ps*	partial actin domain with 111 aa
4	DDB0237450	*act36_ps*	partial actin domain with 135 aa
5	DDB0237454	*act37_ps*	partial actin domain with 67 aa
6	DDB0237455	*act38_ps*	partial actin domain with 88 aa
7	DDB0238642	*act39_ps*	partial actin domain with 122 aa

#### Phylogeny of the *D. discoideum* actinome

**Figure 2:**
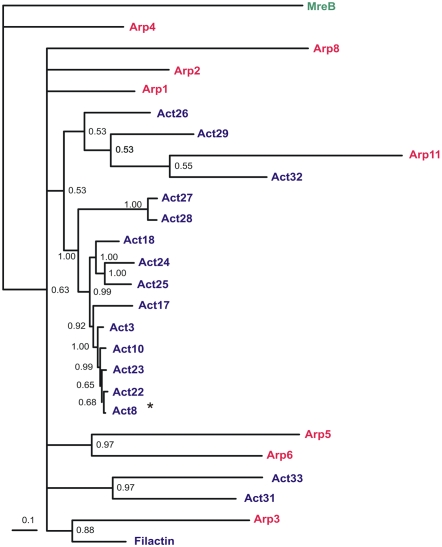
Phylogenetic tree of the *D. discoideum* actinome. Mrbayes 3.12 was used for tree construction and 100,000 trees were generated with a sample frequency of 100 and a total of 1000 trees. Treeview 1.6.6 was used for tree visualization. The scale bar corresponds to the branch length and shows 0.1 amino acid substitution per site. The bootstrap values are shown on the branch forks. The 17 identical actins are not listed separately and represented only by Act8 (*). In constrast to the actin-related proteins (red), the number of mutations in most of the conventional actins (blue) remained relatively low.

**Figure 3:**
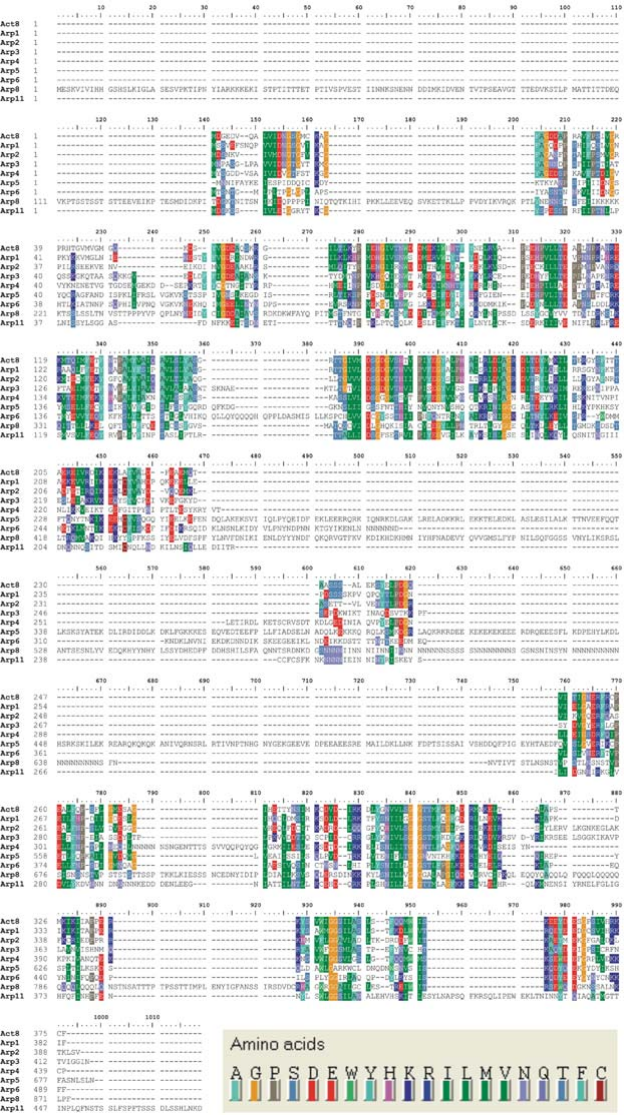
Alignment of *D. discoideum* Arps with Act8. The alignment was done with the programme BioEdit (version 7.0.5.3), the extensive color coding is explained at the lower right. Except Arp1, all other actin related proteins contain additional amino acid stretches that disrupt the conventional actin domain profile. This is especially obvious in Arp4, Arp5, Arp6, Arp8 and Arp11.

The phylogenetic tree of the *D. discoideum* actinome (Fig. 2) shows Arp4 as closest to bacterial actin-like protein MreB, which was used as an outgroup and is thought to be a putative ancestor of all actins [15], [16]. In the tree, Act8 again represents all 17 identical proteins as they are listed in Figure 1. The most closely related actin is Act22 that differs from this group by three amino acid exchanges (A236S, Y280F, A320S). Act10 with one single residue exchange only (D2E) is more distant, which reflects the scores in the permutation matrix used by the alignment program. Exchanges from A>S, Y>F, and D>E score 1, 0, and 3 points, respectively, thus listing Act22 as more closely related to the 17 identical actins than Act10. An alignment of all Arps with the Act8 protein sequence is shown in Fig. 3. Filactin is not included because it belongs to the *bona fide* actins and contains a compact actin domain that is highly homologous to conventional actin.

Eleven of the actin genes that code for the group of 17 identical proteins are located on chromosome 2, four on chromosome 5, and one each on chromosomes 1 and 3 (Table 2). A phylogenetic tree of the DNA sequences from the identical actin proteins clearly shows the series of multiplication events (Fig, 4). Most of the genes are clustered. This suggests a wave of gene duplications especially on chromosome 2. Detailed analysis of the DNA upstream and downstream of the actin genes did not lead to further information about the putative duplications.

**Figure 4:**
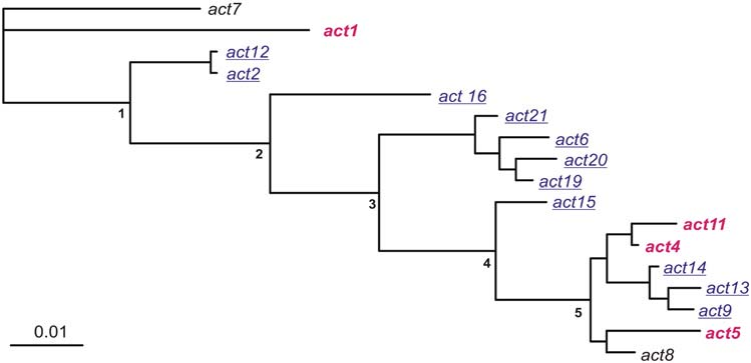
Putative duplications of actin genes. The presence of 17 identical conventional actins, each one encoded by a distinct gene, suggests a series of gene duplications. Most of these genes are clustered on chromosome 2 (blue, underlined) and chromosome 5 (red, bold). An analysis of the corresponding genomic DNA implies 5 consecutive duplication events. The tree was generated with the programme DNAML 3.5c.

**Table 2:** Chromosomal location of 17 actin genes (Act8 goup) that code for identical proteins.

#	Proteins	dictyBase ID	Chromosomal Location
1	Act1	DDB0220444	Chromosome **5** coordinates **2938326** to **2939456**, **Crick** strand
2	Act2	DDB0185124	Chromosome **2** coordinates **4708449** to **4709579**, **Watson** strand
3	Act4	DDB0220448	Chromosome **5** coordinates **2234318** to **2235448**, **Crick** strand
4	Act5	DDB0220447	Chromosome **5** coordinates **3186992** to **3188122**, **Crick** strand
5	Act6	DDB0185126	Chromosome **2** coordinates **4042347** to **4043477**, **Watson** strand
6	Act7	DDB0220445	Chromosome **3** coordinates **3684122** to **3685252**, **Watson** strand
7	Act 8	DDB0216213	Chromosome **1** coordinates **4602557** to **4603687**, **Watson** strand
8	Act9	DDB0220456	Chromosome **2** coordinates **4441895** to **4443025**, **Watson** strand
9	Act11	DDB0220449	Chromosome **5** coordinates **2167534** to **2168664**, **Watson** strand
10	Act12	DDB0216214	Chromosome **2** coordinates **4705207** to **4706337**, **Crick** strand
11	Act13	DDB0220454	Chromosome **2** coordinates **4481524** to **4482654**, **Crick** strand
12	Act14	DDB0220455	Chromosome **2** coordinates **4404974** to **4406104**, **Watson** strand
13	Act15	DDB0185015	Chromosome **2** coordinates **1767622** to **1768752**, **Watson** strand
14	Act16	DDB0185127	Chromosome **2** coordinates **1594273** to **1595403**, **Crick** strand
15	Act19	DDB0220446	Chromosome **2** coordinates **4040120** to **4041250**, **Watson** strand
16	Act20	DDB0220450	Chromosome **2** coordinates **4034200** to **4035330**, **Crick** strand
17	Act21	DDB0220451	Chromosome **2** coordinates **4038493** to **4039623**, **Crick** strand

A sequence homology search of the human genome using the most typical *D. discoideum* actin (Act8) as query sequence finds the major actin isoforms such as cytoplasmic beta (ACTB) and gamma actins (ACTG), aortic smooth muscle actin (ACTA), and alpha cardiac muscle actin (ACTC) as best hits. This was also the case with a reciprocal search. At a first glance, the data suggest that the sequence of conventional actins reached a nearly perfect evolutionary level in *D. discoideum*, which changed only marginally during further evolution to higher eukaryotes. However, if one compares the still limited data from the *D. fasciculatum* genome with the *D. discoideum* actinome, the family expansion seems to have occurred independently (see below). This renders it unlikely that one can directly correlate the evolution of amoeba and human actins despite their similarities.

#### Characteristic structural features in the actin molecule

Actins contain five highly conserved sequence motifs that include the adenosine-binding loop (adeno), two phosphate-binding loops (ph-1 and ph-2), and two connecting domains (con-1 and con-2). These sequences are conserved in conventional actins and in serveral Arps, but only to a small extent in structural homologues like hexokinase, the Hsp70 family, other sugar kinases and prokaryotic cell cycle proteins such as MreB, FtsA and StbA [17].

**Figure 5:**
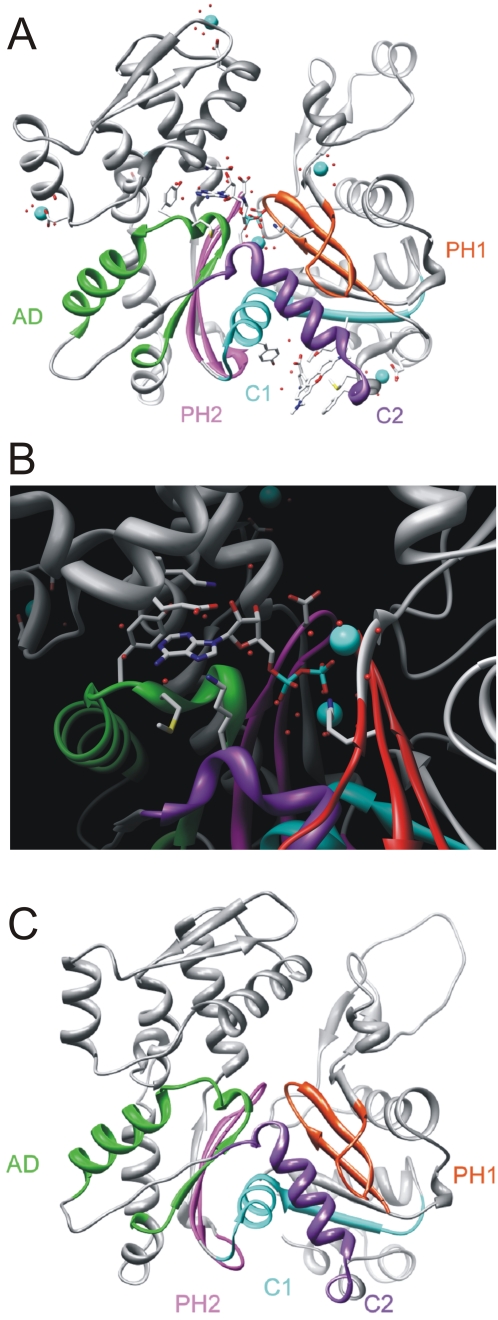
Actin crystal structure, ATP-binding motifs, and modeled Act8 structure. (A) The ribbon model of muscle actin (PDB ID: 1J6Z) shows the characteristic structure of four subdomains (1–4), the topology of the binding motifs, the wireframe of ADP, calcium ions (blue) and water molecules (red). (B) Five highly conserved sequences line the nucleotide binding pocket: the adenosine binding loop AD (green), the two phosphate binding loops PH1 and PH2 (red and purple, respectively) and the subdomain connecting motifs C1 and C2 (blue and violet, respectively). These sequences are highly conserved signatures in actins and actin-related proteins. (C) Model of Act8, the representative of the 17 identical actins in *D. discoideum*.

**Figure 6:**
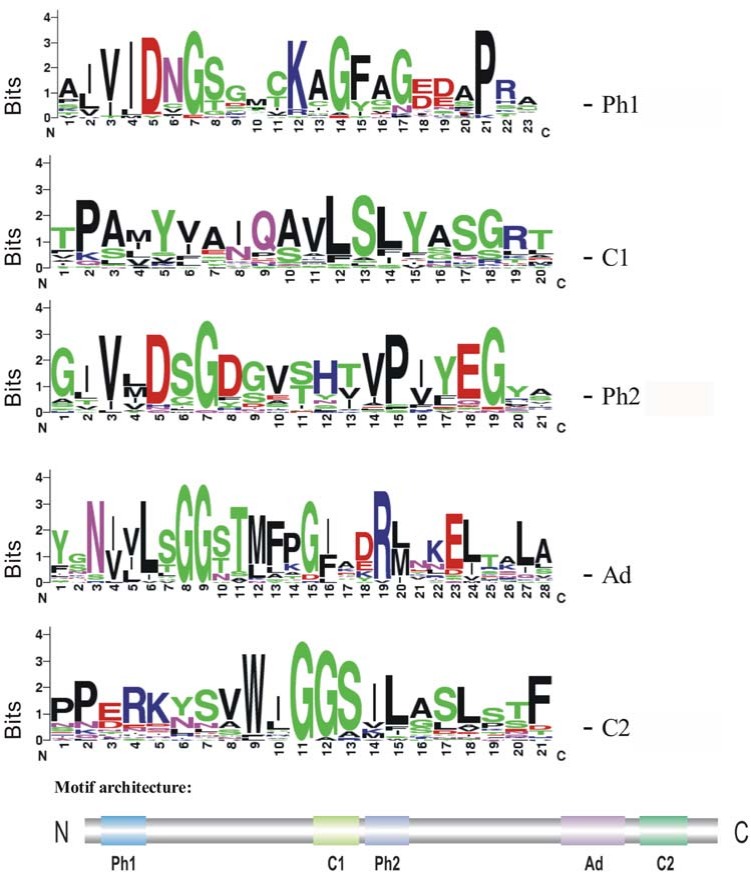
Motif logos from *D. discoideum* actins and Arps. The five structural motifs of all members of the *D. discoideum* actinome are summarized as sequence logos which reflect the structural, functional and evolutionary significance of specific amino acids at a particular position. It is remarkable that there is a surprisingly pronounced variability of conservation and that only three glycine residues are seemingly indispensable in all 41 members of the *D. discoideum* actinome (# 8 and #9 in the adeno domain, # 11 in Con-2).

The five structural motifs of human beta actin (ACTB) were taken to classify all members of the *D. discoideum* actinome as actins or actin-related proteins. Figure 5A/B shows a ribbon model of rabbit muscle actin and as an enlargement the topology of the structural motifs that interact with the adenosine-moiety (green), the beta- (purple) and gamma-phosphates (red). The three-dimensional orientation of these motifs is essential for ATP binding and hydrolysis. In Fig. 5C the representative *D. discoideum* Act8 was modeled into the crystal structure and shows an excellent agreement with the known actin fold. Using this motif architecture we compared all actins and actin-related proteins of the *D. discoideum* actinome and screened for shared and therefore likely essential amino acids in the actin structure. Fig. 6 shows sequence logos [18] where the presence of a conserved amino acid at a particular position reflects its structural, functional and evolutionary significance. Absolutely conserved residues are surprisingly rare. The data suggest that the actin profiles in *D. discoideum* are highly variable in their possible interactions with other molecules and, consequently, in their individual functions.

**Figure 7:**
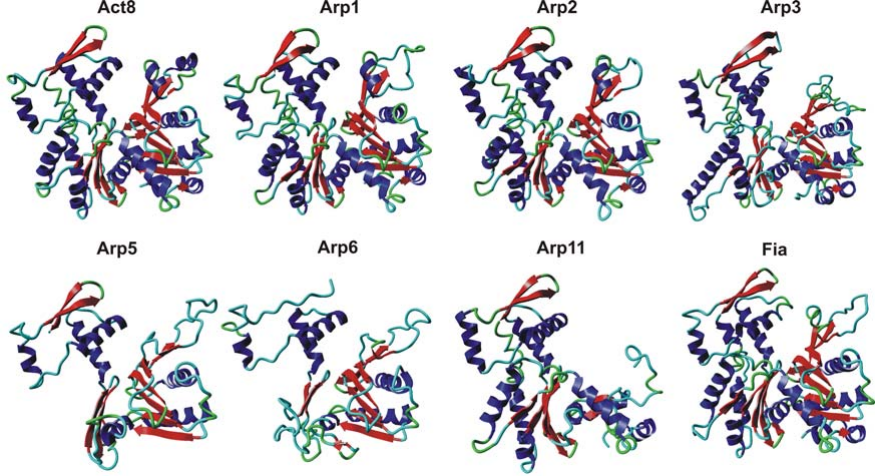
Structural homology among the *D. discoideum* Arps. The sequences were modeled in comparison to the Act8-type actin (upper left). Whereas Arps 1, 2 and 3 show high similarity to the three-dimensional structure of actin, Arp 5, 6 and 11 are clearly different. Attempts to model Arps 4 and 8 failed due to low structural homology and the absence of available templates. Filactin (Fia, lower right) shows high structural homology in the actin domain. Modeling was done using the Swiss Model Server [58], graphics were generated with the YASARA molecular visualization software [59].

A structural analysis of the actin-related proteins shows drastic differences of their putative folds (Fig. 7). Of course one has to be careful with this type of computerized modeling. But as it was already stated before [3], the term ‘actin-related’ is certainly misleading and reflects rather sequence than functional similarities. Only Arps 1, 2 and 3 are highly similar to normal Act8. This was also shown in a comparison of similarities in the amino acid sequences [8]. Among the putative structures of Arp 5, 6 and 11 only the latter one shows in subdomains 3 and 4 some similarity to conventional actin. This agrees with the position of Arp 11 in Fig. 2. *D. discoideum* Arp8 contains large inserts in the conserved motifs PH1 and C2 (see Fig. 5). Thus, the structure would be distorted in the essential nucleotide binding region. Not surprisingly, Arp8 was not detected in the cytoskeleton but found to be a subunit in INO80, a large chromatin remodeling complex [19]. The *D. discoideum* Arp4 and Arp8 protein sequences are so divergent that the software did not find a template even at lowest stringency. Therefore, the structures are not included in Fig. 7. In this report Filactin (Fia) is classified as an actin because its actin domain is extremely homologous to conventional actin.

#### Comparative analysis of the *D. discoideum* and *D. fasciculatum* actinomes

**Figure 8:**
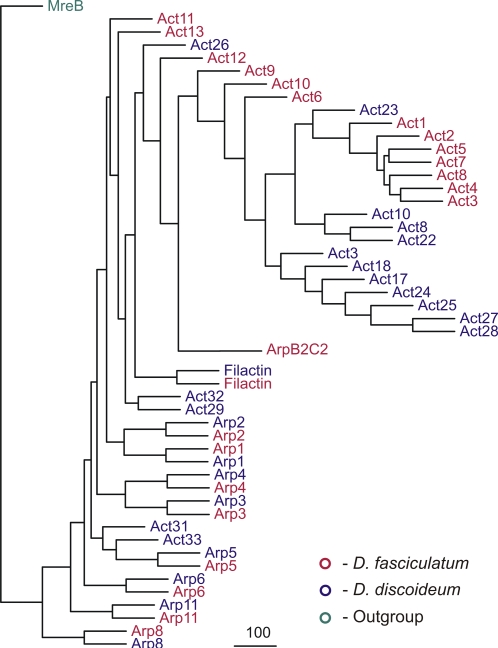
Phylogenetic comparison of the *D. discoideum* (blue) and *D. fasciculatum* (red) actinomes. Both genomes code for many conventional actins which, however, form distinct groups. In contrast, the Arps from both organisms are in all cases closely related and form branched pairs.

Upon completion of the entire *D. discoideum* genome [10] comparative genomics now allow detailed and very specific studies on distinct protein families in closely related organisms. Based on a number of characteristics including the comparison of small subunit ribosomal RNA and α-tubulin protein sequences the social amoebae can be divided into four evolutionary groups [11], [12]. *D. discoideum* belongs to the furthest developed group 4. The species in this group have larger fruiting bodies and spores, their cAMP oscillations are more sensitive and occur earlier in development. This allows recruitment of more cells from a wider catchment area to the emerging aggregates. A current comparative genome project includes social amoebae from the other three groups. The most advanced analysis comes from *D. fasciculatum*, a member of the most ancient group 1. In the meantime, the whole genome is shotgun sequenced to a 18-fold coverage (Gernot Glöckner, unpublished) and allows preliminary comparisons of genes that code for actin and actin-related proteins. Both the genome size and the gene density are similar to that of *D. discoideum*. Apparently there is no long range synteny in the genomes of these two organisms, but *D. fasciculatum* contains numerous actin genes and all the Arps (Fig. 8). Interestingly, there is a high and consistent homology between the Arps from these two species. Arp1, 2, 3, 4, 5, 6, 8 and 11 team up as pairs in the tree. In contrast, the actins form distinct species-specific groups.

#### Expression patterns of actin isoforms

**Figure 9:**
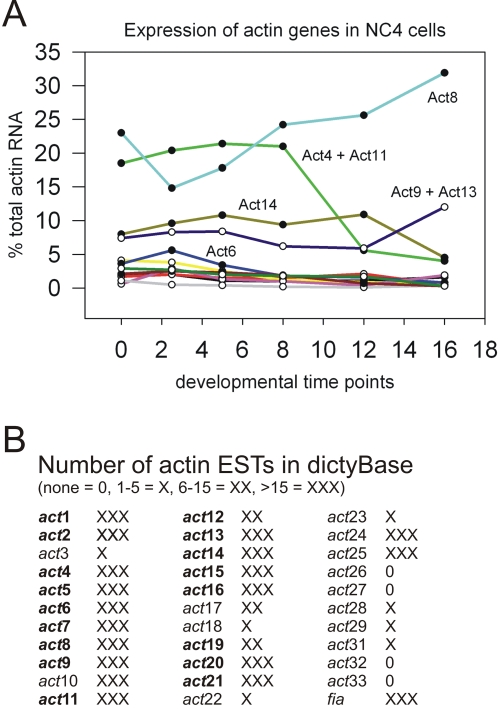
Expression of actin genes. (A) The expression of individual actin genes in vegetative and developing *D. discoideum* NC4 cells was analyzed 1985 by Firtel and coworkers with gene-specific probes [26]. The nomenclature of the actin genes was now updated and the tabular values of actins from the Act8 group were taken for the graph in the upper panel. Act8 mRNA is present throughout development and at high concentration. Act4 and Act11 mRNAs have been analyzed together and followed a distinct developmental pattern. Among the lower concentrated mRNAs only Act6, Act9, Act13 and Act14 mRNAs reach levels above 5% of total actin RNA. The graph shows that essentially all genes from the Act8 group are transcribed. (B) Also the number of identified ESTs reflects mRNA concentrations from specific genes. The actinome ESTs listed in dictyBase were counted and grouped according to their numbers. The genes from the Act8 group are shown in bold and all of them have a large or very large number of ESTs.

Conventional actins are in general extremely abundant proteins. However, this does not exclude expression of specific actin isoforms at low concentrations, in distinct subcellular regions, or for a short time during development only. Data are still sparse but the activities of actin genes in *D. discoideum* shed some light onto intriguing expression patterns. It has been known for quite some time that the classical Act8-type isoform represents more than 95% of total actin in the amoeba [20]. These data were obtained by protein sequencing of purified actin samples and it was, of course, at that time unknown whether one, a few or all of the 17 *act8*-type genes contributed to the overall actin pool in a *D. discoideum* cell. An approach with antibodies is not possible because all *act8*-type genes code for identical proteins. It turned out that ‘pre-genomic’ experiments by Firtel and coworkers more than 20 years ago are now an excellent source to analyze the expression patterns of actin genes [21], [22], [23], [24], [25]. Total actin mRNA was purified from different developmental stages and selectively quantified in Northern blots using specific DNA probes from the upstream or downstream non-coding regions. Fig. 9A shows a graph which is based on data published by Romans et al., 1985 [26]. The authors found that *act8* (Act8 group) is expressed at high levels throughout growth and development. The large abundance of *act8* mRNA was not only found in the strain NC4, but also in the axenic laboratory strain Ax3 [27]. Recent microarray data essentially confirm the expression patterns of actin genes during development [28].

The presence and number of actin-specific ESTs reflects in a rough estimation the transcription activity of the corresponding genes as well. Fig. 9B quantifies the ESTs (none = 0 ESTs, low number (X) = 1–5 ESTs, medium number (XX) = 6–15 ESTs, high number (XXX)>15 ESTs) as they are currently listed in dictyBase [29]. According to these data all 17 *act8*-type genes are expressed in the most abundant EST group (XXX = >15), with the small exception of *act12* and *act19* (EST group XX = 6–15). Therefore, probably all genes from the *act8*-group contribute to the >95% pool of identical actin protein.

The gene *act8* which generates the highest mRNA levels is located on chromosome 1 and does not belong to an actin gene cluster (Fig. 4). Furthermore, if one compares Firtel's data on actin mRNA levels (Fig. 9A) with the localization of *act8*-type genes on individual chromosomes and their putative multiplication during evolution (Fig. 4) one can assume that the duplications included the promoter regions. Expression of the genes *act9* and *act13* has been tested in Northern blots with 5′- and even more specific 3′-probes: both genes follow the same expression pattern during development. In the tree in Fig. 4 *act9* and *act13* appear as pairs. The same might be true for the genes *act4* and *act11* (Fig. 4, Fig. 9A).

**Figure 10:**
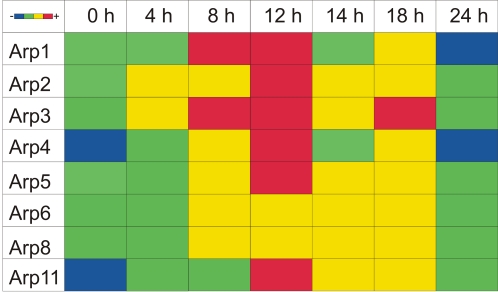
Expression of Arp genes during development as determined by a realtime PCR approach. The mRNA concentrations are color-coded from low (blue) to high (red) levels. It remains to be shown whether the relatively high concentrations during development are mirrored by the corresponding protein levels and specific activities.

The number of ESTs (Fig. 9B) sheds light also onto the importance of actin isoforms for distinct cellular functions. Four genes (*act26*, *act27*, *act32*, *act33*) might not be transcribed at all, might be expressed with only minute activities, or are transcribed for a short time only at distinct developmental stages. The actin genes with only a very small amount of ESTs (X = 1–5) do not belong to the conventional *act8*-group and have, most likely, rather regulatory than structural functions. It is consistent with this assumption, that overexpression of the unconventional actin isoform Act3 alters the normal actin network (own unpublished observations). The expression patterns of the genes coding for Arp isoforms vary during development as well, as analyzed by quantitative PCR (Fig 10). However, it requires more biochemical and cell biological data to correlate the expression patterns of distinct Arp genes with their protein functions during *D. discoideum* development.

#### Localization signals and other motifs

Nuclear localization signals (NLS) are short regions of mainly basic amino acids [30]. The PredictNLS server clearly identified *D. discoideum* Arp5 (NLS: KKKQR QLKSM KDGRL AQKRKR) and Arp8 (NLS: RKKKEK) as putative nuclear proteins. This correlates well with recent reports that identified these two Arps as members of chromatin remodeling complexes [19], [31]. On the other hand, nuclear export signals (NES) are characterized by distinctly spaced hydrophobic amino acids and are essential for the export of a protein out of the nucleus. These signals were predicted for the *D. discoideum* actinome using the NetNES server [32]; the results from the server are, however, not as detailed as they could be collected from the work of E. Nishida and coworkers [33]. Therefore, we included in Table 3 both NES predictions for the *D. discoideum* actinome (Table 3). We also found that none of the members of the actinome contain a predictable signal peptide or a transmembrane helix as analyzed by SignalP [34] and TMHMM [35].

**Table 3:** Putative nuclear export signals.

#	Proteins	Signals predicted using the NetNES 1.1 server	Signals predicted based on Wada et al. 1998 [33]
			(top: NES1, bottom: NES2)
1	Act3	176-ILR**L**D**L**AGRD**L**TDY-189	171-S**L**PHA**I**LR**L**D**L**AG-183
		219-YVA**L**DFE-225	212-D**I**KEK**L**AY**V**A**L**D-223
2	Act10	176-ILR**L**D**L**AGRD**L**TDY-189	171-A**L**PHA**I**LR**L**D**L**AG-183
		219-YVA**L**DFE-225	212-D**I**KEK**L**AY**V**A**L**D-223
3	Act8*	176-ILR**L**D**L**AGRD**L**TDY-189	171-A**L**PHA**I**LR**L**D**L**AG-183
		219-YVA**L**DFE-225	212-D**I**KEK**L**AY**V**A**L**D-223
4	Act17	216-KLS**Y**IT**L**DFQ-225	171-S**I**NHA**I**SR**L**D**L**AG-183
			212-D**I**KEK**L**SY**I**T**L**D-223
5	Act18	178-RLD**L**AGRD**L**SDY-189	171-A**L**PHA**I**LR**L**D**L**AG-183
		219-YVA**L**DFD-225	212-D**I**KEK**L**SY**V**A**L**D-223
6	Act22	176-ILR**L**D**L**AGRD**L**TDY-189	171-A**L**PHA**I**LR**L**DLAG-183
		219-YVA**L**DFE-225	212-D**I**KEK**L**AY**V**A**L**D-223
7	Act23	160-ILR**L**D**L**AGRD**L**TDY-173	155-A**L**PHA**I**LR**L**D**L**AG-163
		203-YVA**L**DFE-209	196-D**I**KEK**L**AY**V**A**L**D-207
8	Act24	178-RLH**L**AGG-184	171-TFPLS**I**TR**L**H**L**AG-183
		220-YVA**L**DFE-226	213-D**I**KEK**L**AY**V**A**L**D-224
9	Act25	190-ILS**L**D**L**AGR-199	186-I**L**RQA**I**LS**L**D**L**AG-198
		234-YVA**L**DFE-240	227-G**I**KEK**L**SY**V**A**L**D-238
10	Act26	218-YVA**I**NFN-224	170-V**L**QHS**I**IR**L**NFAG-182
			211-S**I**KEK**L**AY**V**A**I**N-222
11	Act27	NIL	160-A**L**PHATSS**L**GFAG-172
			197-D**I**KEK**L**SY**V**SSD-208
12	Act28	NIL	162-A**L**PLATSS**L**GFAG-174
			199-D**I**KEK**L**SY**V**SSD-210
13	Act29	NIL	177-S**I**PNS**I**NQ**L**E**I**AG-189
			219-D**I**KEKFGF**I**S**L**N-230
14	Filactin	20-RLV**L**HKN-25	739-S**I**PHA**I**KR**I**D**I**GG-751
			780-D**I**KEKTSF**V**SQD-791
15	Arp1	287-DMS**I**RKS-293	174-A**L**PHA**I**SR**I**D**I**AG-186
			215-I**I**KEKTCY**V**AHD-226
16	Arp2	NIL	172-S**I**PHLTRR**L**D**V**AG-184
			213-Q**I**KEK**L**CY**V**AYD-224
17	Arp3	57-DLD**F**FIG-63	185-V**I**GSS**I**KH**I**P**I**AG-197
			226-R**V**KEQYSY**V**CPD-237
18	Arp4	NIL	186-V**V**KNG**I**VKSN**L**AG-198
			(SYKRYVTLET**I**R)
19	Arp5	323-ILA**L**KTT-329	284-RQRKIQNRKD**L**GA-296
			(T**L**KSKSYATEKD)
20	Arp6	425-KER**LELEL**RKL-435	212-R**L**NYA**I**KRFN**I**GG-224
			251-T**I**KEKTCF**I**SKD-262
21	Arp8	334-TLL**L**KEL-340	384-L**L**PNTRLT**L**GYGG-396
			(DFSDNIKIENLD)
22	Arp11	80-KES**L**FIF-86	170-G**I**LKAYKS**I**S**L**GS-182
			219-Q**L**LNDKILNS**I**Q-230
23	Act 31 (355aa)	NIL	164-P**V**TDA**V**VT**L**DFGG-176
			205-Q**I**KEKHSF**I**E**L**D-216
24	Act 32(392aa)	227-YVS**L**NYN-233	179-I**I**TKAMSH**L**P**L**GG-191
			220-D**I**KEK**L**GY**V**S**L**N-231
25	Act33 (414aa)	360-RLK**I**ELG-366	190-L**L**KEG**I**VRQEFGG-202
			NIL

The signals according to Wada et al. (1998) have been identified manually. Sequences in brackets had too weak characteristics to define them as explicit nuclear export signals.

#### Protein stability

PEST motifs (rich in the amino acids P, E, S and T) reduce the half-lives of proteins dramatically and target them for proteolytic degradation [36], [37]. Table 4 contains a list of proteins from the actinome that harbor PEST motifs. Among them are the centrosome-specific Arp1 (centractin), the putative subunits from nuclear complexes (Arp5, Arp8), and filactin. The latter is a conventional actin with a long N-terminal extension. Filactin is notoriously difficult to purify due to its biochemical instability (unpublished observations).

**Table 4:** PEST motifs in members of the *D. discoideum* actinome as analyzed by PESTfind.

#	Proteins	PEST Motifs
1	Filactin	KESTATIDQFPSPPTSNISTTSTTTTTT
2	Arp1	KEEELLEPDSSSS
3	Arp5	KGEEVEDPEEAEES
4	Arp8	KIDVENTVTPSEAVGTTTEDV
		KPTSSTSSTSTTEEVEI
5	Arp11	KEITSDNETITTTNQIPT

### II. Actinomes of other model organisms

#### Conventional actins

**Figure 11:**
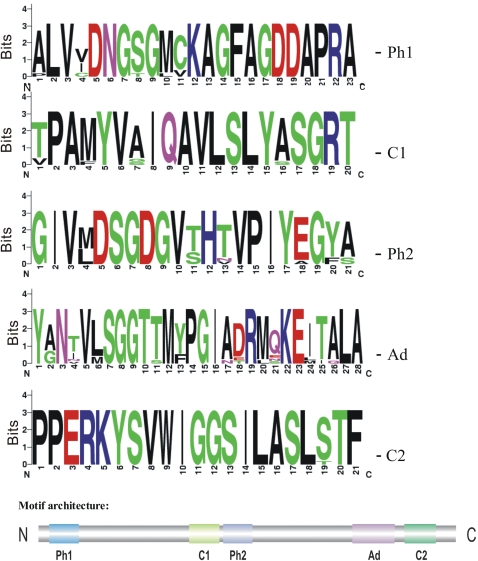
Motif logos from Act8 homologues across species. The five structural actin motifs are summarized as sequence logos which reflect the structural, functional and evolutionary significance of specific amino acids at a particular position. In difference to the variability of the motif profiles in *D. discoideum* only (see Fig. 6), the homologies in conventional actins throughout evolution are extremely high. The following actins have been compared: *Arabidopsis thaliana*, Q541W9_ARATH - *Caenorhabditis elegans*, ACT2_CAEEL - *Dictyostelium discoideum*, Act8, DDB0216213 - *Drosophila melanogaster*, ACT1_DROME(Act5C) - *Entamoeba histolytica*, Q51B76_ENTHI - *Gallus gallus*, ACTB_CHICK (beta-actin) - *Homo sapiens*, ACTG_HUMAN (gamma-actin) - *Mus musculus*, ACTG_MOUSE (gamma-actin) - *Saccharomyces cerevisiae*, ACT_YEAST - *Takifugu rubripes*, ACT1_FUGRU, (beta-actin1) - *Xenopus laevis*, ACTB_XENLA (beta-actin).

The evolutionary relationship of *D. discoideum* actin (Act8-type) across species was studied in a blast search for best hits in genomes of model organisms (Table 5) and with the MrBayes software [38]. However, one should be aware that the extreme similarities of actins throughout all eukaryotic species complicate analysis with today's available software. In addition, incomplete taxon sampling or presumed accelerated evolution disturb topologies as well. The high similarities of the actin fold across species lead to conserved motif logos (Fig. 11). Conventional actins throughout evolution were apparently under huge pressure towards structure and function.

**Table 5:** Act8 from *D. discoideum* was used as a query sequence in a blast comparison with the genomes from ten other prominent model organisms.

*#*	Organism	Protein ID	Blast Scores (Bits)	E value	Best hit *(isoform)*	Uniprot ID
1	*E. histolytica* [60]	Eh_ACT	695	0.0	actin	P11426
2	*S. cerevisiae* [61]	Sc_ACT1	686	0.0	actin	P60010
3	*A. thaliana* [62], [63]	At_ACT11	712	0.0	actin	P53496
4	*C. elegans* [64]	Ce_ACT-2	724	0.0	actin	P10984
5	*D. melanogaster* [65]	Dm_ACT5C	724	0.0	actin	P10987
6	*X. laevis*	Xl_ACTB	725	0.0	beta-actin	O93400
7	*T. rubripes*	Tr_ACTB-A	723	0.0	beta-actin	P68142
8	*G. gallus* [66]	GG_ACTG1	724	0.0	gamma-actin	Q5ZMQ2
		Gg_ACTB	724	0.0		P60706
9	*M. musculus* [67], [68]	Mm_ACTG1	724	0.0	gamma-actin	P63260
		Mm_ACTB	724	0.0		P60710
10	*H. sapiens* [69]	Hs_ACTG1	724	0.0	gamma-actin	P63261
		Hs_ACTG2	724	0.0		P63267
		Hs_ACTB	724	0.0		P60709

The best hits and their scores show extreme homologies even to mammalian actins.

#### Actin-related proteins

The Arp families were classified in *S. cerevisiae* as Arp1 to Arp10 based on the descending order of their sequence similarity to actin [39]. The additional Arp11 was then identified in human [40]. Apart from this, there is a family of orphan Arps. The *S. cerevisiae* genome encodes all the Arps but Arp11, while Arp7, Arp9 and Arp10 are specific to yeast. Most organisms contain single copy Arp genes, with the exception of *E. histolytica*, which has two genes that encode an Arp5 [41]. The *X. laevis* genome contains two Arp2 isoforms [41] and a few yeast species have two copies of Arp4 [8]. With just four Arps (Arp2, 3, 4, 5) the parasite *E. histolytica* has only a small repertoire of actin-related proteins. However, the host cell might compensate for the absence of other ubiquitous Arps. *T. rubripes* has the most compact genome, which contains only two Arp genes encoding Arp3 and Arp5, so far the smallest number of actin-related proteins in a sequenced genome (Table 6).

**Table 6:** Occurrence of Arps in model organisms. (* assembly).

#	Organisms	Arp1	Arp2	Arp3	Arp4	Arp5	Arp6	Arp7	Arp8	Arp9	Arp10	Arp11
1	*A. thaliana* [62], [63]	**−**	**+**	**+**	**+**	**+**	**+**	**−**	**+**	**−**	**−**	**−**
2	*E. histolytica* [60]	**−**	**+**	**+**	**+**	**+**	**−**	**−**	**−**	**−**	**−**	**−**
3	*D. discoideum* [10], [29]	**+**	**+**	**+**	**+**	**+**	**+**	**−**	**+**	**−**	**−**	**+**
4	*S. cerevisiae* [61]	**+**	**+**	**+**	**+**	**+**	**+**	**+**	**+**	**+**	**+**	**−**
5	*D. melanogaster* [65]	**+**	**+**	**+**	**+**	**+**	**+**	**−**	**+**	**−**	**−**	**+**
6	*C. elegans* [64]	**+**	**+**	**+**	**+**	**−**	**+**	**−**	**−**	**−**	**−**	**+**
7	* *X. laevis*	**+**	**+**	**+**	**+**	**+**	**−**	**−**	**+**	**−**	**−**	**+**
8	* *T. rubripes*	**−**	**−**	**+**	**−**	**+**	**−**	**−**	**−**	**−**	**−**	**−**
9	* *G. gallus* [66]	**+**	**+**	**+**	**+**	**+**	**+**	**−**	**+**	**−**	**−**	**+**
10	*M. musculus* [67], [68]	**+**	**+**	**+**	**+**	**+**	**+**	**−**	**+**	**−**	**−**	**+**
11	*H. sapiens* [69]	**+**	**+**	**+**	**+**	**+**	**+**	**−**	**+**	**−**	**−**	**+**

#### The multitude of actin genes allows adaptation to environmental changes

The complex actinome of *D. discoideum* is a paradigm for studies on the regulatory function of actin isoforms during development or cell movement. Despite the high number of actin genes, in a protein preparation more than 95% of total actin in the amoeba consist of only one sequence variant [20]. The intriguing presence of a large number of identical actins raises the question why evolution would allow this seemingly luxurious feature. An energy consuming process of keeping 17 distinct genes with identical gene products would be eliminated very quickly during harsh environmental conditions. Only a selective advantage argues against elimination of redundant genes. This type of selection in an actinome might be based on co- or posttranslational modifications that happen only at a specific time during development or in distinct subcellular regions. A number of reports describe post-translational modifications of actin in *D. discoideum*. There are acetylated and nonacetylated actins in a *D. discoideum* homogenate [42], [43], actin can be acylated in vivo with palmitic acid [44], under certain environmental conditions actin is reversibly tyrosine-phosphorylated [45], [46], [47], [48]. Especially during spore formation in late development tyrosine-phosphorylated actin forms bundles and tubes which are disintegrated only at the onset of renewed germination [49], [50], [51], [52], [53], [54]. In a recent report, Korn and coworkers showed that *D. discoideum* actin that was phosphorylated at Tyr-53 had an increased critical concentration, a greatly reduced rate of polymerization and a negligible nucleation activity [55]. Therefore, timely presence and correct localization of the appropriate tyrosine kinase might trigger this putatively co-translational modification. In such a case a tightly regulated expression would guarantee a highly efficient modification which thus is responsible for a strongly selective pressure.

## Materials and Methods

### Computational analyses

The proteins of the actinome were identified from the sequence profiles derived from the Pfam dataset [13]. For construction of phylogenetic trees the sequences were aligned using clustalw 1.83 [56]. The alignments were refined manually in accordance with the conserved region. The trees were generated with the Bayesian method implemented in the software package MrBayes 3.1.2 [38], MreB was set as outgroup, four chains and two runs were done for one million generations. Trees were sampled every hundred generations and the consensus tree was estimated by using a burn in of 3,000 trees. The tree graphics were done with the help of Tree view 1.6.6 [57]. The multiple alignments from the five structural motifs in actin were plotted using WebLogo [18]. WebLogo is a web-based application that generates sequence logos. Sequence logos are a graphical representation of an amino acid or nucleic acid multiple sequence alignment. Each logo consists of stacks of symbols, one stack for each position in the sequence. The overall height of the stack indicates the sequence conservation at that position, while the height of symbols within the stack indicates the relative frequency of each amino or nucleic acid.

The stability of the members of the actinome was assayed using the algorithm PESTfind [36], [37]. The PEST hypothesis was based on a literature survey that combined both information on protein stability and protein primary sequence information. Initially, the study relied on 12 short-lived proteins with well-known properties, but was continually extended later. Although all these proteins exerted different cellular functions it became apparent that they shared high local concentrations of the amino acids proline (**P**), glutamic acid (**E**), serine (**S**), threonine (**T**) and to a lesser extent, aspartic acid (**D**). From that it was concluded that PEST motifs reduce the half-lives of proteins dramatically and hence, that they are target proteins for proteolytic degradation.

The *D. discoideum* actinome was subjected to signal peptide identification using the SignalP 3.0 server [34]. The method incorporates a prediction of cleavage sites and a signal peptide/non-signal peptide prediction; based on machine learning approaches, like neuronal network and hidden Markov model algorithms. The search for transmembrane regions was done using the Transmembrane Hidden Markov Model (TMHMM, [35]). This method predicts transmembrane helices with 97–98% accuracy and can also discriminate between soluble and membrane proteins. PredictNLS [30] is an automated tool for the analysis and determination of nuclear localization signals (NLS). NetNES 1.1 [32] was used to predict leucine-rich nuclear export signals (NES) in eukaryotic proteins using a combination of neuronal networks and hidden Markov models. The sequence data were mostly derived from the NCBI genome database, in a few cases from the databases corresponding to the specific, organisms. At servers where the genes were not listed, they were obtained by using blast software.

### Experimental procedures

RNA isolation and quantification: Total RNA was extracted from *D. discoideum* Ax2 cells at different developmental stages using the Qiagen RNeasy Mini kit. The manufacturer's protocol for the isolation of RNA from the cytoplasm of animal cells was used for preparation. The RNA samples were taken for reverse transcription for RT-PCR (Real-Time PCR) experiments. cDNA was generated using the M-MLV reverse transcriptase, RNAse H minus (Roche) according to the manufacturer's protocol. Usually 1–5 microgram of the respective total RNA was used for each RT reaction. cDNAs generated were used as a template to carry out PCR with the respective gene specific primers. Primers were chosen using the program at http://frodo.wi.mit.edu/cgi-bin/primer3/primer3_www.cgi.

## References

[1] Pantaloni D, Le Clainche C, Carlier MF (2001). Mechanism of actin-based motility.. Science.

[2] Pollard TD, Borisy GG (2003). Cellular motility driven by assembly and disassembly of actin filaments.. Cell.

[3] Schleicher M, Jockusch BM (2008). Actin: its cumbersome pilgrimage through cellular compartments.. Histochem Cell Biol.

[4] McKinney EC, Meagher RB (1998). Members of the Arabidopsis actin gene family are widely dispersed in the genome.. Genetics.

[5] Swiss-Prot http://expasy.org/.

[6] McHugh KM, Crawford K, Lessard JL (1991). A comprehensive analysis of the developmental and tissue-specific expression of the isoactin multigene family in the rat.. Dev Biol.

[7] Vandekerckhove J, Weber K (1978). At least six different actins are expressed in a higher mammal: an analysis based on the amino acid sequence of the amino-terminal tryptic peptide.. J Mol Biol.

[8] Muller J, Oma Y, Vallar L, Friederich E, Poch O (2005). Sequence and comparative genomic analysis of actin-related proteins.. Mol Biol Cell.

[9] Kessin RH (2006). The Secret Lives of Dictyostelium.. Dictyostelium discoideum Protocols.

[10] Eichinger L, Pachebat JA, Glockner G, Rajandream MA, Sucgang R (2005). The genome of the social amoeba Dictyostelium discoideum.. Nature.

[11] Schaap P (2007). Evolution of size and pattern in the social amoebas.. Bioessays.

[12] Schaap P, Winckler T, Nelson M, Alvarez-Curto E, Elgie B (2006). Molecular phylogeny and evolution of morphology in the social amoebas.. Science.

[13] Finn RD, Mistry J, Schuster-Bockler B, Griffiths-Jones S, Hollich V (2006). Pfam: clans, web tools and services.. Nucleic Acids Res.

[14] Dictybase http://dictybase.org/.

[15] Carballido-Lopez R (2006). The bacterial actin-like cytoskeleton.. Microbiol Mol Biol Rev.

[16] Rivero F, Cvrckova F (2007). Origins and evolution of the actin cytoskeleton.. Adv Exp Med Biol.

[17] Bork P, Sander C, Valencia A (1992). An ATPase domain common to prokaryotic cell cycle proteins, sugar kinases, actin, and hsp70 heat shock proteins.. Proc Natl Acad Sci U S A.

[18] Crooks GE, Hon G, Chandonia JM, Brenner SE (2004). WebLogo: a sequence logo generator.. Genome Res.

[19] Chen M, Shen X (2007). Nuclear actin and actin-related proteins in chromatin dynamics.. Curr Opin Cell Biol.

[20] Vandekerckhove J, Weber K (1980). Vegetative Dictyostelium cells containing 17 actin genes express a single major actin.. Nature.

[21] Firtel RA (1981). Multigene families encoding actin and tubulin.. Cell.

[22] Firtel RA, Timm R, Kimmel AR, McKeown M (1979). Unusual nucleotide sequences at the 5′ end of actin genes in Dictyostelium discoideum.. Proc Natl Acad Sci U S A.

[23] McKeown M, Taylor WC, Kindle KL, Firtel RA, Bender W (1978). Multiple, heterogeneous actin genes in Dictyostelium.. Cell.

[24] Romans P, Firtel RA (1985). Organization of the actin multigene family of Dictyostelium discoideum and analysis of variability in the protein coding regions.. J Mol Biol.

[25] Romans P, Firtel RA (1985). Organization of the Dictyostelium discoideum actin multigene family. Flanking sequences show subfamily homologies and unusual dyad symmetries.. J Mol Biol.

[26] Romans P, Firtel RA, Saxe CL (1985). Gene-specific expression of the actin multigene family of Dictyostelium discoideum.. J Mol Biol.

[27] McKeown M, Firtel RA (1981). Differential expression and 5′ end mapping of actin genes in Dictyostelium.. Cell.

[28] Booth EO, Van Driessche N, Zhuchenko O, Kuspa A, Shaulsky G (2005). Microarray phenotyping in Dictyostelium reveals a regulon of chemotaxis genes.. Bioinformatics.

[29] Urushihara H, Morio T, Saito T, Kohara Y, Koriki E (2004). Analyses of cDNAs from growth and slug stages of Dictyostelium discoideum.. Nucleic Acids Res.

[30] Cokol M, Nair R, Rost B (2000). Finding nuclear localization signals.. EMBO Rep.

[31] Shen X, Ranallo R, Choi E, Wu C (2003). Involvement of actin-related proteins in ATP-dependent chromatin remodeling.. Mol Cell.

[32] la Cour T, Kiemer L, Molgaard A, Gupta R, Skriver K (2004). Analysis and prediction of leucine-rich nuclear export signals.. Protein Eng Des Sel.

[33] Wada A, Fukuda M, Mishima M, Nishida E (1998). Nuclear export of actin: a novel mechanism regulating the subcellular localization of a major cytoskeletal protein.. Embo J.

[34] Nielsen H, Engelbrecht J, Brunak S, von Heijne G (1997). A neural network method for identification of prokaryotic and eukaryotic signal peptides and prediction of their cleavage sites.. Int J Neural Syst.

[35] Krogh A, Larsson B, von Heijne G, Sonnhammer EL (2001). Predicting transmembrane protein topology with a hidden Markov model: application to complete genomes.. J Mol Biol.

[36] Rechsteiner M, Rogers SW (1996). PEST sequences and regulation by proteolysis.. Trends Biochem Sci.

[37] Rogers S, Wells R, Rechsteiner M (1986). Amino acid sequences common to rapidly degraded proteins: the PEST hypothesis.. Science.

[38] Ronquist F, Huelsenbeck JP (2003). MrBayes 3: Bayesian phylogenetic inference under mixed models.. Bioinformatics.

[39] Boyer LA, Peterson CL (2000). Actin-related proteins (Arps): conformational switches for chromatin-remodeling machines?. Bioessays.

[40] Frankel S, Mooseker MS (1996). The actin-related proteins.. Curr Opin Cell Biol.

[41] NCBI-Database.

[42] Rubenstein P, Smith P, Deuchler J, Redman K (1981). NH2-terminal acetylation of Dictyostelium discoideum actin in a cell-free protein-synthesizing system.. J Biol Chem.

[43] Rubenstein P, Deuchler J (1979). Acetylated and nonacetylated actins in Dictyostelium discoideum.. J Biol Chem.

[44] Stadler J, Gerisch G, Bauer G, Deppert W (1985). In vivo acylation of Dictyostelium actin with palmitic acid.. Embo J.

[45] Jungbluth A, Eckerskorn C, Gerisch G, Lottspeich F, Stocker S (1995). Stress-induced tyrosine phosphorylation of actin in Dictyostelium cells and localization of the phosphorylation site to tyrosine-53 adjacent to the DNase I binding loop.. FEBS Lett.

[46] Jungbluth A, von Arnim V, Biegelmann E, Humbel B, Schweiger A (1994). Strong increase in the tyrosine phosphorylation of actin upon inhibition of oxidative phosphorylation: correlation with reversible rearrangements in the actin skeleton of Dictyostelium cells.. J Cell Sci.

[47] Howard PK, Sefton BM, Firtel RA (1993). Tyrosine phosphorylation of actin in Dictyostelium associated with cell-shape changes.. Science.

[48] Schweiger A, Mihalache O, Ecke M, Gerisch G (1992). Stage-specific tyrosine phosphorylation of actin in Dictyostelium discoideum cells.. J Cell Sci.

[49] Kishi Y, Clements C, Mahadeo DC, Cotter DA, Sameshima M (1998). High levels of actin tyrosine phosphorylation: correlation with the dormant state of Dictyostelium spores.. J Cell Sci.

[50] Kishi Y, Mahadeo D, Cervi DN, Clements C, Cotter DA (2000). Glucose-induced pathways for actin tyrosine dephosphorylation during Dictyostelium spore germination.. Exp Cell Res.

[51] Sameshima M, Chijiiwa Y, Kishi Y, Hashimoto Y (1994). Novel actin rods appeared in spores of Dictyostelium discoideum.. Cell Struct Funct.

[52] Sameshima M, Kishi Y, Osumi M, Mahadeo D, Cotter DA (2000). Novel actin cytoskeleton: actin tubules.. Cell Struct Funct.

[53] Sameshima M, Kishi Y, Osumi M, Mahadeo D, Cotter DA (2002). Electron microscopy of actin rods and bundles in Dictyostelium discoideum by high-pressure freezing.. J Electron Microsc (Tokyo).

[54] Sameshima M, Kishi Y, Osumi M, Minamikawa-Tachino R, Mahadeo D (2001). The formation of actin rods composed of actin tubules in Dictyostelium discoideum spores.. J Struct Biol.

[55] Liu X, Shu S, Hong MS, Levine RL, Korn ED (2006). Phosphorylation of actin Tyr-53 inhibits filament nucleation and elongation and destabilizes filaments.. Proc Natl Acad Sci U S A.

[56] Thompson JD, Higgins DG, Gibson TJ (1994). CLUSTAL W: improving the sensitivity of progressive multiple sequence alignment through sequence weighting, position-specific gap penalties and weight matrix choice.. Nucleic Acids Res.

[57] Page RD (1996). TreeView: an application to display phylogenetic trees on personal computers.. Comput Appl Biosci.

[58] Schwede T, Kopp J, Guex N, Peitsch MC (2003). SWISS-MODEL: An automated protein homology-modeling server.. Nucleic Acids Res.

[59] Kalay E, Uzumcu A, Krieger E, Caylan R, Uyguner O (2007). MYO15A (DFNB3) mutations in Turkish hearing loss families and functional modeling of a novel motor domain mutation.. Am J Med Genet A.

[60] Loftus B, Anderson I, Davies R, Alsmark UC, Samuelson J (2005). The genome of the protist parasite Entamoeba histolytica.. Nature.

[61] Hirschman JE, Balakrishnan R, Christie KR, Costanzo MC, Dwight SS (2006). Genome Snapshot: a new resource at the Saccharomyces Genome Database (SGD) presenting an overview of the Saccharomyces cerevisiae genome.. Nucleic Acids Res.

[62] Initiative AG (2000). Analysis of the genome sequence of the flowering plant Arabidopsis thaliana.. Nature.

[63] Seki M, Narusaka M, Kamiya A, Ishida J, Satou M (2002). Functional annotation of a full-length Arabidopsis cDNA collection.. Science.

[64] Consortium CeS (1998). Genome sequence of the nematode C. elegans: a platform for investigating biology.. Science.

[65] Davis MB, White KP (2004). Recent advances in Drosophila genomics.. Genome Biol.

[66] Consortium ICGS (2004). Sequence and comparative analysis of the chicken genome provide unique perspectives on vertebrate evolution.. Nature.

[67] Strausberg RL, Feingold EA, Grouse LH, Derge JG, Klausner RD (2002). Generation and initial analysis of more than 15,000 full-length human and mouse cDNA sequences.. Proc Natl Acad Sci U S A.

[68] Waterston RH, Lindblad-Toh K, Birney E, Rogers J, Abril JF (2002). Initial sequencing and comparative analysis of the mouse genome.. Nature.

[69] Consortium IHGS (2004). Finishing the euchromatic sequence of the human genome.. Nature.

